# Generation of IL-8 and IL-9 Producing CD4^+^ T Cells Is Affected by Th17 Polarizing Conditions and AHR Ligands

**DOI:** 10.1155/2014/182549

**Published:** 2014-02-20

**Authors:** Michaela Gasch, Tina Goroll, Mario Bauer, Denise Hinz, Nicole Schütze, Tobias Polte, Dörthe Kesper, Jan C. Simon, Jörg Hackermüller, Irina Lehmann, Gunda Herberth

**Affiliations:** ^1^UFZ-Helmholtz Centre for Environmental Research Leipzig, Department of Environmental Immunology, Permoserstrasse, 04318 Leipzig, Germany; ^2^Junior Research Group Immune Pathogenesis of New Allergies, Leipzig Research Centre for Civilization Diseases, University of Leipzig, Johannisallee, 04103 Leipzig, Germany; ^3^Institute for Laboratory Medicine and Pathobiochemistry, Philipps University of Marburg, Hans-Meerwein Strasse, 35043 Marburg, Germany; ^4^Universitätsklinikum, Klinik für Dermatologie, Venerologie und Allergologie, Philipp-Rosenthal Strasse, 04103 Leipzig, Germany; ^5^Young Investigators Group Bioinformatics and Transcriptomics, Department of Proteomics, UFZ-Helmholtz Centre for Environmental Research, Permoserstrasse, 04318 Leipzig, Germany

## Abstract

The T helper cell subsets Th1, Th2, Th17, and Treg play an important role in immune cell homeostasis, in host defense, and in immunological disorders. Recently, much attention has been paid to Th17 cells which seem to play an important role in the early phase of the adoptive immune response and autoimmune disease. When generating Th17 cells under *in vitro* conditions the amount of IL-17A producing cells hardly exceeds 20% while the nature of the remaining T cells is poorly characterized. As engagement of the aryl hydrocarbon receptor (AHR) has also been postulated to modulate the differentiation of T helper cells into Th17 cells with regard to the IL-17A expression we ask how far do Th17 polarizing conditions in combination with ligand induced AHR activation have an effect on the production of other T helper cell cytokines. We found that a high proportion of T helper cells cultured under Th17 polarizing conditions are IL-8 and IL-9 single producing cells and that AHR activation results in an upregulation of IL-8 and a downregulation of IL-9 production. Thus, we have identified IL-8 and IL-9 producing T helper cells which are subject to regulation by the engagement of the AHR.

## 1. Introduction

Th17 differentiation conditions are often used to simulate a chronic inflammatory situation *in vitro* with the aim to unravel the underlying mechanism of related autoimmune diseases like multiple sclerosis, rheumatoid arthritis, or psoriasis (reviewed in [1]). Therefore, the analysis of the orchestration of T helper (Th) cell subtypes generated under these conditions is essential not only for understanding the process of disease development but also for the establishment of therapeutic approaches. By now, IL-17A, IFN-*γ*, IL-9, and IL-22 producing T cells have been described to be generated under Th17 polarizing conditions [2, 3]. Nevertheless, the percentage of IL-17A^+^ or IL-9^+^ cells generated under Th17 polarizing conditions is very low ranging from 2 to 20% [3, 4] and the nature of other CD4^+^ T cell subsets coming up under these conditions has not been further elucidated. An important role in inflammation is attributed to IL-8 (CXCL8), a pleiotropic chemokine with a high neutrophil-attracting capacity (reviewed in [5]). However, as IL-8 is not a typical T helper cytokine, it has not been investigated intensively in T cell differentiation assays.

In models of autoimmune diseases such as experimental autoimmune encephalomyelitis (EAE) it became evident that the aryl hydrocarbon receptor (AHR) plays an important role in regulation of the immune response [6, 7]. AHR activation by its ligand 2,3,7,8-Tetrachlorodibenzo-p-dioxin (TCDD) induced functional Treg, whereas AHR activation by 6-Formylindolo[3,2-b]carbazole (FICZ) boosted Th17 cell differentiation and increased the severity of EAE in mice [6]. In humans TCDD and FICZ have been shown to enhance IL-22 while simultaneously decreasing IL-17A production by CD4^+^ T cells [8, 9]. However, the effect of AHR ligands on CD4^+^ T cells producing IL-8 and IL-9 known to also be involved in inflammation has not been analyzed yet. In addition, the effect of the AHR ligand benzo[a]pyrene (B[a]P), a component of cigarette smoke [10], on CD4^+^ T cells is still unknown. Furthermore, the involvement of miR-326, which is positively correlated with IL-17A expression of human CD4^+^ T cells [11], is not yet elucidated in AHR-mediated immune regulation and may offer new aspects in this process. Thus, in the present study we aimed to investigate changes at cellular, protein, as well as mRNA and microRNA level in CD4^+^ T cells challenged with AHR ligands (TCDD, FICZ, and B[a]P) under Th17 polarizing conditions (TGF-*β*, IL-1*β*, and IL-23). We observed that Th17 polarizing conditions and AHR ligands not only affect IL-17A production but mainly influence the generation of IL-8 and IL-9 producing CD4^+^ T cells. In addition, we show here that the AHR ligand FICZ influences the expression of miR-326 in a time-dependent manner, which might lead to an altered IL-17A production in human CD4^+^ T cells.

## 2. Methods

### 2.1. Human CD4^+^ T Cell Isolation, Cell Culture, and Differentiation

Buffy Coats were obtained from healthy human donors (Institute of Transfusion Medicine, University of Leipzig, Germany) with fully informed written consent, conducted in accordance with the Declaration of Helsinki and approved by the Ethics Committee of the University of Leipzig (272-12-13082012). Peripheral blood mononuclear cells (PBMC) were purified by Ficoll density gradient centrifugation (Ficoll Paque PLUS, GE Healthcare Europe GmbH, Freiburg, Germany) and CD4^+^ T helper cells (purity > 96%) were isolated by negative selection using CD4^+^ T cell isolation Kit II (Miltenyi Biotec, Bergisch Gladbach, Germany). CD4^+^ T cells were cultured in IMDM + GlutaMAX supplemented with 10% KnockOut Serum Replacement (both Invitrogen, Life Technologies, Carlsbad, CA, USA) and 5 × 10^−5 ^M *β*-Mercaptoethanol (Merck, Darmstadt, Germany) at 37°C in 5% CO_2_ humidified air. The cells were stimulated with plate bound anti-CD3 (1.5 *μ*g/mL; UCHT1) and soluble anti-CD28 antibodies (1 *μ*g/mL; CD28.2; both BD Bioscience, Heidelberg, Germany) and for Th17 polarizing conditions recombinant human proteins (TGF-*β*1 (0.5 ng/mL), IL-23 (10 ng/mL, both R&D Systems, Minneapolis, MN, USA); IL-1*β* (10 ng/mL, ebioscience, Frankfurt, Germany)) were added. CD4^+^ T cells were cultured in the presence or absence of the AHR-ligands 6-Formylindolo[3,2-b]carbazole (FICZ, 100 nM, ENZO Life Sciences, Lörrach, Germany), 2,3,7,8-Tetrachlorodibenzo-p-dioxin (TCDD, 5 nM, LGC Standards GmbH, Wesel, Germany), or Benzo-[a]-pyrene (B[a]P, 100 nM, Sigma Aldrich, St. Louis, MO, USA) dissolved in DMSO (Sigma Aldrich). DMSO (0.05%) was used as a solvent control. Supernatants were harvested every day in a 5-day culture period and stored at −80°C for further cytokine measurement. Cultured cells were harvested at indicated time points for extracellular and intracellular staining and flow cytometry as well as for protein and total RNA isolation.

### 2.2. Flow Cytometry

For intracellular cytokine staining cultured cells were restimulated with PMA (50 ng/mL, Sigma Aldrich) and Ionomycin (1 *μ*g/mL, Invitrogen, Life technologies) in the presence of Monensin (2.5 mM, Sigma Aldrich) for 5 h. To measure the cell viability (>80%) the cells were stained with Annexin-V FITC and 7-AAD according to the manufacturer's protocol (BD Bioscience). The cells were fixed with 4% paraformaldehyde and permeabilized with 0.1% saponin-containing buffer before washing and staining in different combinations with anti-CD4 (Beckman Coulter, Krefeld, Germany), anti-IL-17A, anti-IL-22, anti-IL-9, anti-IL-8, anti-AHR, anti-ROR*γ*t (ebioscience), and anti-IFN-*γ* (BD Bioscience). All dotplots and histograms shown refer to gated CD4^+^ T cells. Data were acquired on FACSCantoII (BD Bioscience) and were analyzed with FlowJo Software (Tree Star Inc., Ashland, OR, USA).

### 2.3. Cytokine Measurement in Cell Culture Supernatant

Culture supernatants were analyzed for IL-17A, IL-9, IL-8, and IFN-*γ* by flow cytometry using BD CBA Human Soluble Flex Set System (BD Bioscience) according to the manufacturer's instructions and as described previously [12]. In brief, cytokine specific antibody coated beads were incubated for 1 hour with 25 *μ*L of supernatants or standard solution. Thereafter samples were incubated with the corresponding PE labelled detection antibodies for 2 hours. After one washing step samples were measured by flow cytometry. Analysis of data and quantification of cytokines was performed using the FCAP Array software (BD Bioscience) on the basis of corresponding standard curves. IL-22 concentrations were measured by ELISA using the Human IL-22 ELISA Ready-SET-Go!-Kit (ebioscience) according to the manufacturer's instructions.

### 2.4. Western Blot

Total protein of cultured cells was isolated with peqGOLD TriFast (Peqlab, Erlangen, Germany) according to the manufacturer's protocol. Protein concentrations were measured using DC Protein Assay according to the manufacturer's instructions (Bio-Rad Laboratories Munich, Germany). Proteins (5–10 *μ*g) were separated under denaturing conditions on 4–12% NuPAGE Bis-Tris gradient gels (Invitrogen, Life Technologies) and transferred onto nitrocellulose membranes (Bio-Rad Laboratories). After blocking with ECL prime Blocking Reagent (GE Healthcare Europe GmbH), proteins were detected using anti-AHR (ab 2770, RTP1, abcam, Cambridge, UK) and anti-*β*-Actin (A2228, AC-74, Sigma-Aldrich) as well as horseradish peroxidase-conjugated secondary anti-mouse IgG-specific antibody (Bio-Rad Laboratories). Bands corresponding to the proteins of interest were visualized with ECL Select Western Blotting Detection Kit (GE Healthcare Europe GmbH) and FluorChem 8900 imager (Alpha Innotec, Kasendorf, Germany) and quantified with ImageJ (NIH, Bethesda, MD, USA; rsbweb.nih.gov).

### 2.5. Real-Time PCR for mRNA and microRNA Expression

Total RNA was isolated with peqGOLD TriFast and transcribed into cDNA with ImProm-II-Reverse Transcriptase according the manufacturer's instructions (Promega, Mannheim, Germany). The Biomark HD system with 96.96 Dynamic Array Integrated Fluidic Circuit (IFC; Fluidigm, San Francisco, CA, USA) was used for real-time PCR [13]. Primers were predesigned by Universal ProbeLibrary Assay Design Center (Roche applied science, Mannheim, Germany) and purchased from BioTeZ (Berlin Buch GmbH, Berlin, Germany). UPL probes were purchased from Roche Applied Science. Primer sequences are documented in Table 1. The preamplification PCR was performed on the LightCycler 480 System (Roche Diagnostics GmbH, Mannheim, Germany). Thermal cycling and detection of the reaction products were performed with the Biomark HD system (Fluidigm). The data was analyzed with the BioMark Gene Expression Data Analysis software and the ΔΔCt-method. The data is presented as relative expression to the mean of *β*-D-glucoronidase (GUSB), peptidylprolyl-isomerase-A (PPIA), and phosphoglycerate-kinase1 (PGK1) reference gene expression and to the expression at day 0.

For microRNA (miRNA) expression 100 ng total RNA was reverse-transcribed into cDNA using the miRCURY LNA Universal RT Kit according to the manufacturer's instructions (Exiqon A/S, Vedbaek, Denmark). The miRNA expression was detected by real-time PCR using the miRCURY LNA Universal RT microRNA PCR LNA PCR primer set hsa-miR-326 and the reference gene PCR primer set SNORD44 (hsa) (Exiqon A/S). The PCR was performed in triplicate on the LightCycler 480 System. The expression of miRNA was calculated semiquantitative by the ΔΔCt-method. All results are presented as relative expression to SNORD44 reference gene expression and the miRNA expression at day 0.

### 2.6. Statistical Analysis

The number of independently performed experiments is mentioned in the legends of each figure. Statistical significance was calculated with unpaired Student's *t*-test (Statistica for Windows version 10, [StatSoft Inc. (Europe), Hamburg, Germany]) after log-transformation or with Mann-Whitney-*U* test of the original data. All *P* values <0.05 were considered to be significant.

## 3. Results

### 3.1. Th17 Polarizing Conditions Induce IL-8 and IL-9 Producing CD4^+^ T Cells

**Figure 1:**
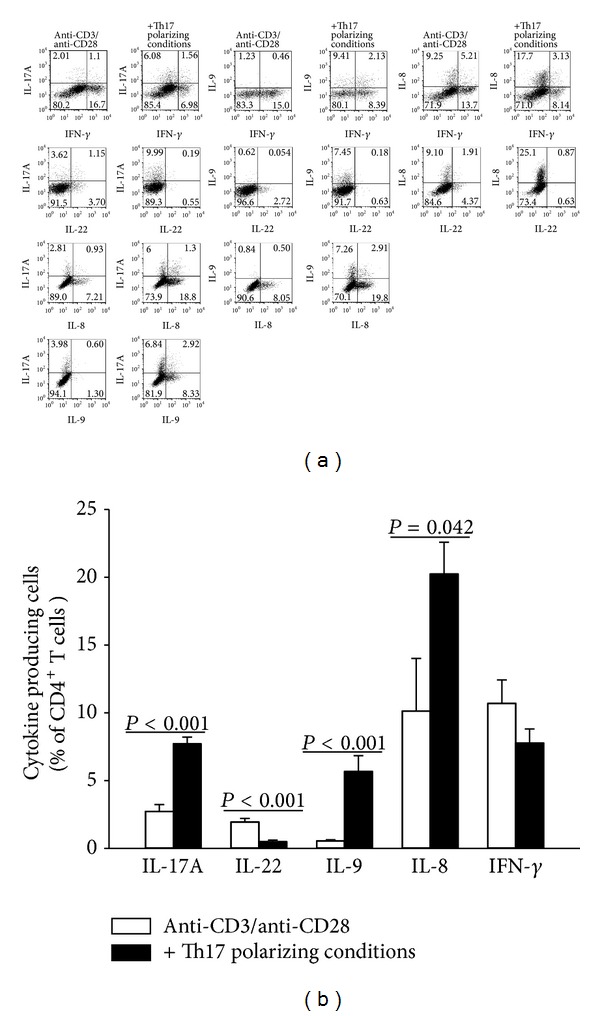
Effect of Th17 polarizing conditions on the T helper cell cytokine production. Human CD4^+^ T cells were cultured with anti-CD3/anti-CD28 in the presence or absence of Th17 polarizing conditions for five days. After restimulation with PMA, Ionomycin, and Monensin for five hours and fixation/permeabilization the cells were stained for IL-17A, IL-22, IL-9, IL-8, and IFN-*γ* and analyzed by flow cytometry. (a) The numbers in the dotplots represent positive cells in %. Data are representative of 3–5 independent experiments. (b) The amount of cytokine producing cells [%] is represented as mean and SEM. Bars with *P* values indicate significant differences between the two groups, Mann-Whitney *U* test, *n* = 5.

**Figure 2:**
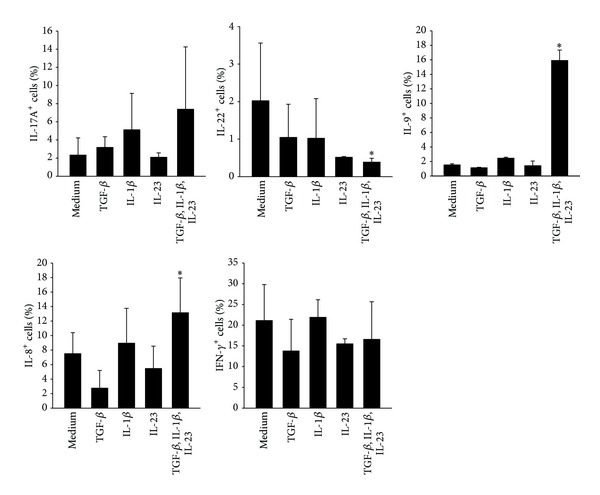
Effect of single cytokine application on the T helper cell cytokine production. Human CD4^+^ T cells were cultured with anti-CD3/anti-CD28 in the presence or absence of the indicated cytokines for 5 days. CD4^+^ T cells cultured with anti-CD3 and anti-CD28 were used as control (medium). After restimulation with PMA, Ionomycin, and Monensin for five hours and fixation/permeabilization the cells were stained for IL-17A, IL-22, IL-9, IL-8, and IFN-*γ* and analyzed by flow cytometry. The amount of cytokine producing cells [%] is represented as mean and SEM, *represents significance compared to control, *P* < 0.05, Mann-Whitney *U* test, and *n* = 3.

For Th17 polarizing conditions the cytokines TGF-*β*, IL-1*β*, and IL-23 were added to anti-CD3/anti-CD28 stimulated human CD4^+^ T cells. After five days of culture cytokine protein expression was analyzed by intracellular staining as well as in cell culture supernatant. CD4^+^ T cells activated by anti-CD3 and anti-CD28 without additional cytokines were used as control. Compared to the control the Th17 polarizing conditions induced, besides IL-17A (from 2.73% ± 0.5 to 7.71% ± 0.49) and IL-9 producing CD4^+^ T cells (from 0.54% ± 0.89 to 5.67% ± 1.17), a high number of IL-8 producing CD4^+^ T cells (from 10.13% ± 1.72 to 20.32% ± 2.43) (Figures 1(a) and 1(b)). In contrast to this, the amount of IL-22 producing cells was reduced in Th17 polarized cells (from 1.95% ± 0.26 to 0.49% ± 0.1). The amount of IFN-*γ* producing T cells was not affected by Th17 polarizing conditions. With reference to the large amount of IL-8^+^ T cells generated under Th17 polarizing conditions (approx. 20% of all CD4^+^ T cells), a high proportion of these cells produced IL-8 only. As it was observed in cultures with single cytokine application the complete Th17 polarizing conditions were necessary for the enhancement of IL-8 expression in CD4^+^ T cells (Figure 2).

**Figure 3:**
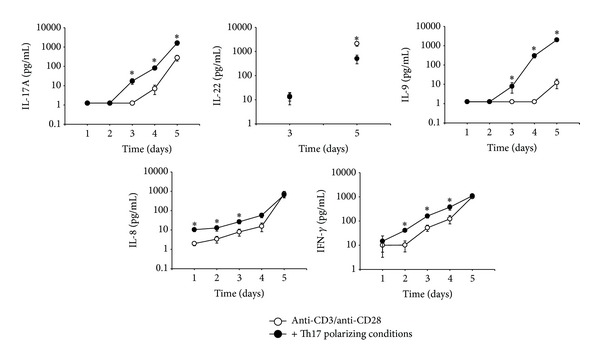
Effect of Th17 polarizing conditions on the cytokine secretion of CD4^+^ T cells. Human CD4^+^ T cells were cultured with anti-CD3/anti-CD28 with or without Th17 polarizing conditions for five days. The concentration of the indicated cytokines in the cell culture supernatant was measured by CBA (IL-17A, IL-9, IL-8, IFN-*γ*) and ELISA (IL-22). The absolute concentration [pg/mL] of IL-17A, IL-22, IL-9, IL-8, and IFN-*γ* in the supernatant of CD4^+^ T cells cultured with anti-CD3/anti-CD28 (○) or in addition of Th17 polarizing conditions (●) during the time course is shown as mean ± SEM. *represents significance, *P* < 0.05, Mann-Whitney *U* test, and *n* = 15.

The measurement of the cytokine concentration in the cell culture supernatant confirmed these results regarding IL-17A, IL-9, and IL-8 induction under Th17 polarizing conditions (Figure 3). Also the secretion of IL-22 was reduced compared to control after five days of culture. It is noteworthy that IL-8 was produced in higher amounts at early time points (approximately 10 pg/mL) compared to IL-17A or IL-9 that were hardly detectable until day three (Figure 3, Table 2). IFN-*γ* secretion was induced by Th17 polarizing cytokines until day 4, but no differences between control and Th17 polarizing conditions were detectable after five days of culture.

**Figure 4:**
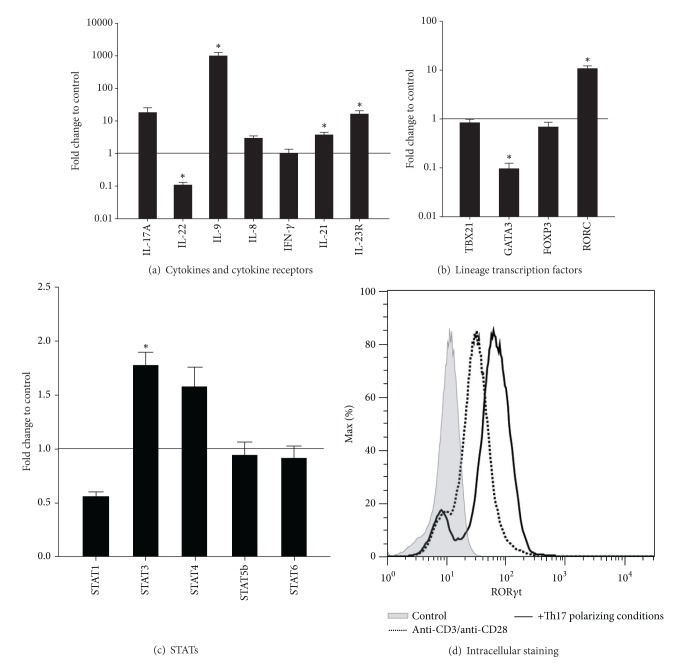
Effect of Th17 polarizing conditions on the mRNA expression and ROR*γ*t protein expression of CD4^+^ T cells. Human CD4^+^ T cells were cultured with anti-CD3/anti-CD28 in the presence or absence of Th17 polarizing conditions for 5 days. (a–c) The mRNA expression of the indicated genes was measured by real-time PCR. Data were normalized to the mean of the reference genes *GUSB, PPIA, PGK1*, and the mRNA expression at day 0 using the ΔΔCt-method. Fold changes in gene expression of CD4^+^ T cells with Th17 polarizing conditions compared to control without Th17 polarizing conditions (black line) are represented as mean and SEM. *represents significance, *P* < 0.05, Mann-Whitney *U* test, and *n* = 7. (d) After restimulation with PMA, Ionomycin and Monensin for five hours and fixation/permeabilization the cells were stained for CD4 and ROR*γ*t and analyzed by flow cytometry. The ROR*γ*t histogram refers to gated CD4^+^ T cells. Unstained cells were used as control, *n* = 2.

The mRNA expression of these cytokines as well as of IL-21 and IL-23R, known to be involved in Th17 polarization, was also regulated by these culture conditions (Figure 4(a)). Furthermore, the mRNA expression of transcription factors, known to be important for Th17 cell function, for example, RORC and STAT3 (reviewed in [2]), was upregulated 10- and 2-fold, respectively (Figures 4(b) and 4(c)). Intracellular ROR*γ*t staining confirmed these results (Figure 4(d)).

### 3.2. Th17 Polarizing Conditions Enhance AHR Expression in CD4^+^ T Cells

The aryl hydrocarbon receptor (AHR) has made its entry into immunology research as a mediator between environment and immune system (reviewed in [14]). We aimed to determine the expression of the AHR in human T helper cells and focused on the influence of different AHR ligands (FICZ, TCDD and B[a]P) on the cytokine expression under Th17 polarizing conditions.

**Figure 5:**
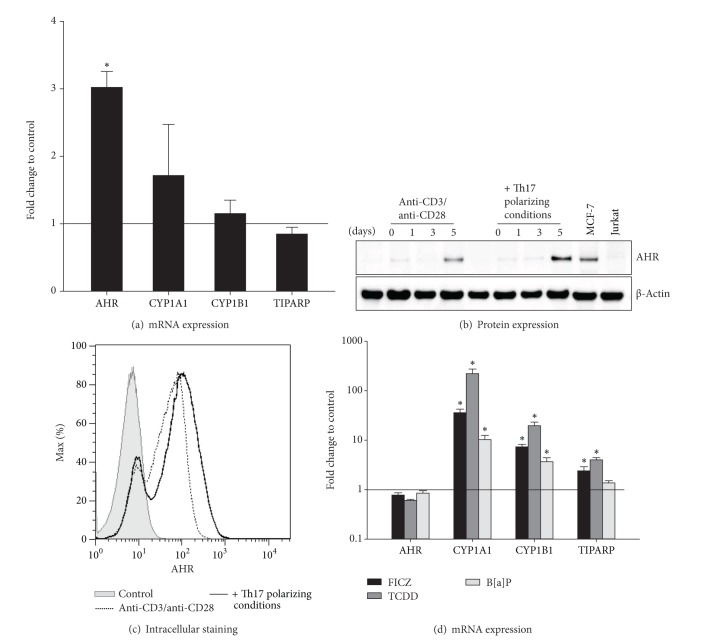
The AHR is induced in CD4^+^ T cells and activated by FICZ, TCDD, and B[a]P. Human CD4^+^ T cells were cultured with anti-CD3/anti-CD28 in the presence or absence of Th17 polarizing conditions for 5 days. (a) The mRNA expression of the indicated genes was measured by real-time PCR. Data were normalized to the mean of the reference genes GUSB, PPIA, PGK1, and the mRNA expression at day 0 using the ΔΔCt-method. Fold changes in gene expression of CD4^+^ T cells with Th17 polarizing conditions compared to control (black line) are represented as mean and SEM. *represents significance, *P* < 0.05, Mann-Whitney *U* test, and *n* = 7. (b) The AHR protein expression (94 kDa) at days 1, 3, and 5 of culture was measured by Western Blot analysis. The protein expression of unstimulated CD4^+^ T cells was used as control. Beta-Actin (42 kDa) antibody was used as loading control. Cell lysates from MCF-7 cell line and Jurkat T cell line were used as AHR positive and negative control, respectively. One representative of 3 independent experiments is shown. (c) After restimulation with PMA, Ionomycin, and Monensin for 5 hours and fixation/permeabilization, the cells were stained for CD4 and AHR and analyzed by flow cytometry. Unstained cells were used as control *n* = 2. (d) The AHR ligands FICZ (100 nM), TCDD (5 nM), and B[a]P (100 nM) were added to CD4^+^ T cells cultured with Th17 polarizing cytokines. CD4^+^ T cells treated with anti-CD3/anti-CD28, Th17 polarizing conditions, and DMSO (0.05%) were used as control. Relative mRNA expression of the indicated genes was measured by real-time PCR and data were normalized to the mean of GUSB, PPIA, and PGK1 and to the mRNA expression at day 0 using the ΔΔCt-method. Data are shown as fold changes of mRNA expression in Th17 polarized and ligand treated CD4^+^ T cells compared to control (black line) and are represented as mean and SEM; *represents significance, *P* < 0.05, Mann-Whitney *U* test, and *n* = 7.

We showed that the AHR mRNA expression was 3-fold upregulated under Th17 polarizing conditions compared to control (Figure 5(a)). In Western Blot analysis the AHR protein was hardly detectable until day five of T cell culture and at this time point Th17 polarized cells showed a higher AHR protein expression compared to control (Figure 5(b)). Intracellular AHR staining confirmed these results (Figure 5(c)). Furthermore, the expression of AHR target genes (CYP1A1, CYP1B1, and TIPARP) was induced after stimulation with the AHR ligands FICZ, TCDD, and B[a]P (Figure 5(d)).

### 3.3. AHR Ligands Enhance IL-8 but Inhibit IL-9 Cytokine Secretion under Th17 Polarizing Conditions

**Figure 6:**
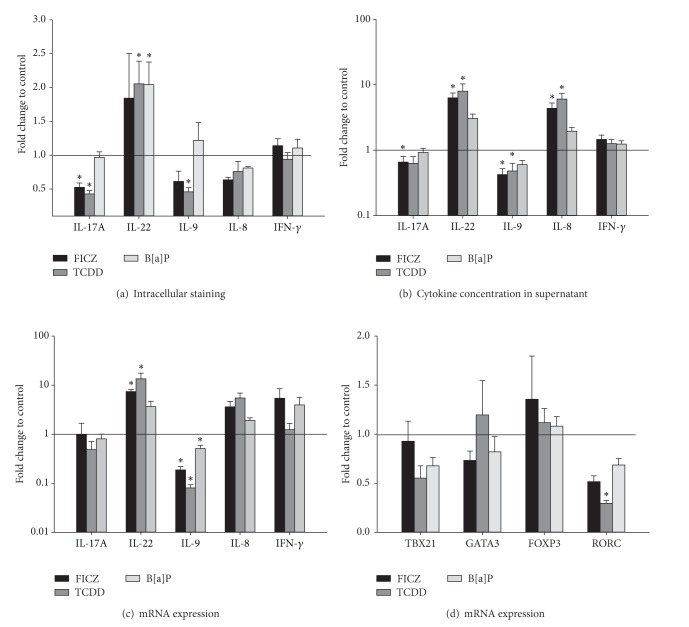
AHR ligands influence the cytokine expression of CD4^+^ T cells. CD4^+^ T cells were cultured with anti-CD3/anti-CD28, Th17 polarizing conditions and the AHR ligands FICZ (100 nM), TCDD (5 nM), and B[a]P (100 nM) for five days. CD4^+^ T cells treated with anti-CD3/anti-CD28, Th17 polarizing conditions, and DMSO (0.05%) were used as control. (a) The amount of cytokine producing cells measured by intracellular staining is represented as fold change to control (black line) and shown as mean and SEM. *represents significance, *P* < 0.05, Mann-Whitney *U* test, and *n* = 5. (b) The concentration of secreted cytokines in the supernatant was measured by CBA (IL-17A, IL-9, IL-8, and IFN-*γ*) and ELISA (IL-22). Data are shown as fold changes of cytokine concentration in the presence of AHR ligands compared to control (black line) and are represented as mean and SEM; *represents significance, *P* < 0.05, Mann-Whitney *U* test, and *n* = 15. (c) and (d) Relative mRNA expression of the indicated genes was measured by real-time PCR and data were normalized to the mean of GUSB, PPIA, and PGK1 and to the mRNA expression at day 0 using the ΔΔCt-method. The fold change of mRNA expression in AHR ligand treated cells compared to control (black line) is represented as mean and SEM; *represents significance, *P* < 0.05, Mann-Whitney *U* test, and *n* = 7.

To investigate the impact of the AHR ligands on the cytokine expression of human CD4^+^ T cells, intracellular as well as secreted proteins were analyzed. The AHR ligands FICZ, TCDD, and B[a]P caused a significant change in the number of cytokine producing CD4^+^ T cells stimulated with anti-CD3/anti-CD28 and Th17 polarizing conditions (Figure 6(a)). Both FICZ and TCDD significantly decreased the amount of IL-17A^+^ T cells by approximately 50%, whereas TCDD also halved the amount of IL-9^+^ T cells. TCDD and B[a]P doubled the proportion of IL-22^+^ T cells.

Regarding the secreted cytokines, FICZ and TCDD significantly increased the concentration of IL-8 and IL-22 at day five of culture compared to control (Figure 6(b)). The concentration of IL-9, strongly induced by Th17 polarizing conditions, was decreased by 50% in FICZ and TCDD treated cell cultures (Figure 6(b)). FICZ also reduced the IL-17A secretion by CD4^+^ T cells. The expression of the Th1 cytokine IFN-*γ* was not affected by the AHR ligands (Figure 6(b) and Table 2). The modification of cytokine secretion occurred in an AHR ligand dependent manner: FICZ and TCDD showed similar results, whereas the addition of B[a]P had no effect.

### 3.4. AHR Ligands Reduce IL-9 and RORC mRNA Expression

At the protein level, the AHR ligands TCDD and FICZ changed the pattern of the analyzed cytokines under Th17 polarizing conditions. Therefore, it was of interest to investigate the underlying molecular mechanisms. The IL-9 mRNA expression was significantly decreased by FICZ, TCDD, and B[a]P under Th17 polarizing conditions (Figure 6(c)). FICZ and TCDD, but not B[a]P, significantly enhanced the IL-22 mRNA expression. These results at least partly confirm the altered cytokine production measured by intracellular staining (Figure 6(a)) and in cell culture supernatant (Figure 6(b)). Furthermore, TCDD decreased the RORC mRNA expression by 70% (Figure 6(d)). RORC is a Th17 specific transcription factor that is essential for IL-17A expression (reviewed in [2]). However, a change in IL-17A mRNA expression was not visible at that time point (Figure 6(c)). In trend also IL-8 mRNA expression was upregulated by FICZ and TCDD compared to control. The expression of the transcription factor STAT3 was not affected by AHR ligands (data not shown).

### 3.5. FICZ Reduces the Expression of miR-326 at Day Three of Culture

**Figure 7:**
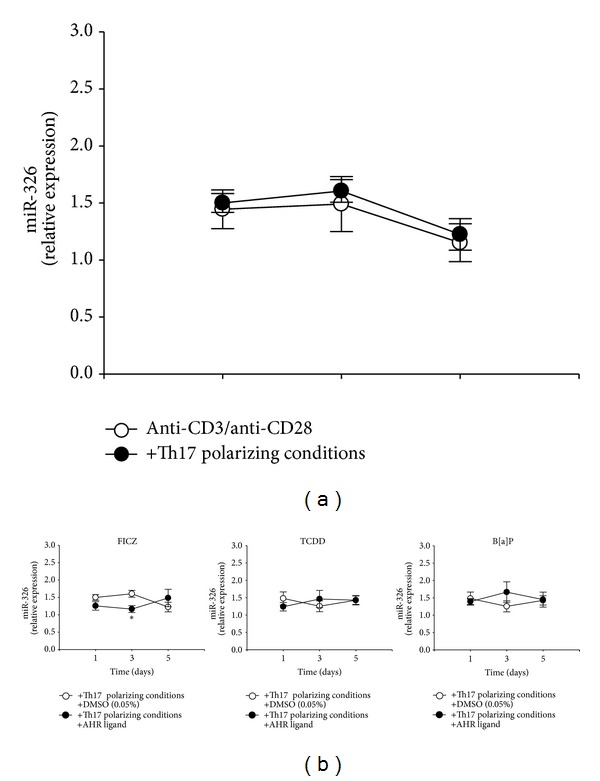
FICZ changes the expression of the Th17 associated microRNA miR-326. (a) The expression of microRNA hsa-miR-326 in CD4^+^ T cells cultured with anti-CD3/anti-CD28 (○) and with Th17 polarizing conditions (●) for 1, 3, and 5 days was measured by real-time PCR. Data were normalized to the expression of hsa-SNORD-44 and the microRNA expression at day 0 using the ΔΔCt-method. Data are shown as mean ± SEM; *represents significance, *P* < 0.05, unpaired Student's *t*-test after log-transformation, and *n* = 5–7. (b) The expression of microRNA hsa-miR-326 in CD4^+^ T cells cultured with anti-CD3/anti-CD28 and Th17 polarizing conditions in the presence (●) of the AHR ligands FICZ (100 nM), TCDD (5 nM), and B[a]P (100 nM) for 1, 3, and 5 days was measured by real-time PCR. CD4^+^ T cells treated with anti-CD3/anti-CD28, Th17 polarizing conditions, and DMSO (0.05%) were used as control (○). Data were normalized to the expression of hsa-SNORD-44 and the microRNA expression at day 0 using the ΔΔCt-method. Data are shown as mean ± SEM; *represents significance, *P* < 0.05, unpaired Student's *t*-test after log-transformation, and *n* = 5–7.

The reduction of IL-17A expression by FICZ and TCDD was observed only on protein but not on mRNA level. To conclude, we aimed to investigate whether factors like miRNAs play a role in this finding. The expression of the Th17 associated microRNA miR-326 was determined by real-time PCR after 1, 3, and 5 days of culture. Unexpectedly, Th17 polarizing conditions did not have any effect on the miR-326 expression at these time points (Figure 7(a)). Regarding the AHR ligands, FICZ significantly downregulated miR-326 expression under Th17 polarizing conditions at day three of culture (Figure 7(b)). B[a]P and TCDD did not influence the expression of miR-326 at any time point. Thus, likewise to the protein and mRNA expression in human CD4^+^ T cells the expression of the Th17 cell related microRNA miR-326 was influenced in an AHR ligand dependent manner.

## 4. Discussion

The discovery of Th17 cells has been followed by the realization that T helper cells can produce various other cytokines alone or in combination in patterns not fitting the preconceived definition of Th1/Th2 or Th17 subsets. These findings had led to the description of additional T helper cell lineages, including Th22 and Th9. In our study Th17 polarizing conditions promoted, besides the known IL-17A, IFN-*γ* and IL-9 producing T helper cells, a distinct CD4^+^ T cell population that produces IL-8 (CXCL8). This cell population mainly expressed IL-8 and coexpressed IL-17A, IL-9, and IFN-*γ* only to a small extent (Figure 1(a)). With measuring the cytokine concentrations in cell culture supernatants it was obvious that IL-8 was strongly induced by Th17 polarizing conditions at the beginning of the T cell culture, whereas the production of cytokines known to be enhanced by Th17 polarizing conditions like IL-17A and IL-9 was not detectable until day three. These findings indicate that the used Th17 polarizing conditions first activate an IL-8 producing T helper cell population. IL-8 is not a typical T helper cell cytokine and by now only two publications have reported IL-8 production in these cells [15, 16]. Schaerli and colleagues reported that approximately 2.5% of CD4^+^ T cells in normal peripheral blood also produce IL-8 [15]. They could show that in neutrophils treated with conditioned medium from IL-8^+^ T cells, apoptosis was reduced by 40%. In lesional skin, IL-8^+^ T helper cells consistently expressed the chemokine receptor CCR6 suggesting that IL-8 producing T cells facilitate skin inflammation by orchestrating neutrophilic infiltration and ensuring neutrophil survival [15]. Recently, Pelletier and colleagues demonstrated that some Th17 T cell clones also produce IL-8, indicating that the chemotactic effect of Th17 cell supernatants on neutrophils can be mainly attributed to IL-8 [16]. In our study the single IL-8 producing as well as the double IL-8^+^/IL-17A^+^ CD4^+^ T cell population increased significantly after culturing with Th17 polarizing conditions. However, the proportion of IL-8^+^/IL-17A^+^ producing cells was very low (1.83% ± 0.78) compared to IL-8^+^/IL-17A^−^ (19.12% ± 5.07) CD4^+^ T cells.

Thus, the question arises whether IL-8 producing T cells generated under Th17 polarizing conditions represent a new effector T cell population or whether it is only a bystander population. Although the presence of low frequencies of IL-8 producing CD4^+^ T cells in the peripheral blood of healthy individuals has been reported years ago [15] a further characterization and chase of these cells has been neglected the following years. The main reason for not paying attention to this cell population and that they remained undiscovered when performing Th17 polarization may lie in the fact that most Th17 experiments are conducted in mice where IL-8 producing T cells do not exist.

Besides IL-17A producing cells also a high proportion of IL-9 producing cells were generated under Th17 polarizing conditions. IL-9^+^ T cells act proinflammatory and are involved in immune mediated diseases ranging from autoimmunity to asthma (reviewed in [17]). IL-17A and IL-9 producing cells have already been placed into central focus of immune therapeutic strategies by the usage of blocking antibodies against the core cytokines of these cells [18, 19]. Certainly, the usage of single antibody treatments does not satisfactorily downregulate inflammatory immune responses. Considering our results that under Th17 polarizing conditions, besides IL-17A^+^ and IL-9^+^ cells, also a substantial proportion of IL-8 producing T cells was generated, it is necessary to also include this cytokine into a therapeutic approach.

As a potent modulator of T cell differentiation the aryl hydrocarbon receptor (AHR) has made its entry into immunology (reviewed in [14, 20]). FICZ and TCDD curtailed IL-17A, IL-9, and IL-8 producing T cells, whereas the number of IL-22 producers was enhanced at day five. B[a]P had a significant effect only on IL-22 producing cells. Cytokine levels in cell culture supernatants stimulated with these ligands partially mirrored the results on cellular level (Figures 6(a) and 6(b)). The finding that the differentiation of IL-17A^+^ and IL-22^+^ T cells is influenced by AHR ligands is in line with previously published data. Ramirez and colleagues demonstrated that TCDD and FICZ reduced IL-17A and enhanced IL-22 production by CD4^+^ T cells under Th17 differentiation conditions [9]. Similar results were gained by the group of Trifari et al. [8]. IL-22 plays a pivotal role in regulating host defense and inflammation (reviewed in [21]) and thereby upregulation of IL-22 production was seen in several human diseases like psoriasis [22, 23], atopic dermatitis [24], and Crohn's disease [25]. Therefore, AHR activation by environmental and endogenous derived ligands may play a role in the induction and process of several inflammatory diseases. New in our findings is that under these conditions also the production of IL-8 and IL-9 is regulated by TCDD and FICZ. We found that both AHR ligands reduced the concentration of IL-9, which is strongly induced by Th17 polarizing conditions, but enhanced the production of IL-8 in the supernatant. Thus, FICZ and TCDD seem to reverse the effects induced by Th17 polarizing conditions except IL-8. Contrarily, B[a]P did not show any effect on cytokine production. Other research groups investigated the impact of AHR ligands on the IL-8 expression in human epidermal keratinocytes (HEK) [26, 27]. BaP was shown to induce IL-8 production in HEK cells [26], whereas FICZ alone was not able to trigger IL-8 production in these cells [27]. However, in this publication FICZ synergizes the effect of resveratrol enhancing IL-8 production in HEK cells. Differences to our data might be explained by the different cell types used as well as by different experimental settings. In our approach cells were not starved and IL-8 measurements were performed at day 5 (compared to 24 hours) and different FICZ concentrations (100 nM versus 1 *μ*M) were used. Furthermore, in contrast to keratinocytes our CD4^+^ T cells were cultured under activation conditions. The Th17 polarizing conditions and the AHR ligands modulated the expression of a variety of cytokines, known to be regulated by different transcription factors and signaling pathways. One reason for this broad effect might be the involvement of microRNAs. Recently, also the AHR pathway was found to be associated with the expression profile of immune relevant microRNAs [28]. Therefore, we aimed to investigate if the expression of miR-326 is modulated by Th17 polarizing conditions and AHR ligands. MiR-326 plays a role in multiple sclerosis models, influencing the IL-17A expression [11, 29]. However, our data reveal that changes at microRNA level were independent of differentiation conditions. In our study, the addition of the AHR ligand FICZ significantly downregulated miR-326 expression in CD4^+^ T cells. It was shown that miR-326 is important for adequate IL-17A expression [11]. Therefore, the inhibitory effect of FICZ on the IL-17A expression might be elicited due to modulation of miR-326 expression. This effect has been observed only at day three of culture. It has been reported that a large fraction of protein-coding genes are under microRNA control and that the amount and the time of microRNA expression are crucial for cell homeostasis or disease development [30]. In contrast to FICZ, TCDD and B[a]P showed no effect on the miR-326 expression

Contrary to the fact that AHR ligands activate the receptor in a similar manner, they cause different effects on the T cell's cytokine, mRNA, and miRNA expression.

Possibly, one explanation is the specific binding affinity of the different ligands to the AHR. The tryptophan derivative FICZ shows an almost ten times higher affinity to the AHR than the prototype ligand TCDD [31], whereas the affinity of the polycyclic aromatic hydrocarbon B[a]P to the AHR is significantly lower compared to FICZ and TCDD (reviewed in [32]).

Another distinguishing feature could be the specific metabolism of the ligands. In this regard, FICZ is degraded very fast after AHR binding [33], whereas TCDD is hardly metabolized and rather accumulates in the body with a half-life of approximately three years [34] leading to a chronic activation of the AHR. Nevertheless, FICZ and TCDD show a different affinity and metabolism; both AHR ligands modulate the cytokine expression of human T helper cells in a similar way. The chronic activation of the AHR by TCDD might compensate its lower receptor affinity. Like FICZ, the AHR ligand B[a]P is also degraded rapidly in an AHR dependent manner [35]. In this study, B[a]P showed only modest effects on the cytokine expression of human T helper cells. Furthermore, the induction of the AHR target genes CYP1A1 and CYP1B1 was lower in B[a]P treated T cells compared to FICZ and TCDD, which indicates a generally weaker AHR activation by B[a]P in human T cells.

Therefore, it can be assumed that the impact of the AHR ligands on the cytokine expression of human T helper cells might be dependent on the ligand affinity as well as the strength and duration of AHR activation.

## 5. Conclusion

In summary, our data reveal that under Th17 polarizing conditions an IL-8^+^/IL-17A^−^/IL-9^−^/IL-22^−^ T helper cell subtype is generated. The proportion of IL-8 producing CD4^+^ T cells was higher than that of other T cell subtypes suggesting a major physiological role of these cells especially under Th17 polarizing conditions. Thus, it will be of great interest to further examine this T cell subtype. Furthermore, IL-9 producing T cells were generated under Th17 polarizing conditions. Both, IL-8 and IL-9 production were affected by the AHR ligands FICZ and TCDD which encourages the opinion of an existing physiological role of the AHR on the human immune system. Moreover, we could show that miR-326 expression was moderately affected by the AHR ligand FICZ. This result shows that besides the classical AHR signalling pathway also the modulation of miRNA expression is potentially involved in the AHR molecular network in human CD4^+^ T cells.

## Figures and Tables

**Table 1:** Primer pairs for real-time PCR analysis.

Gene	Forward primer	Reverse primer
IL-17A	TGGGAAGACCTCATTGGTGT	GGATTTCGTGGGATTGTGAT
IL-9	CAACAAGATGCAGGAAGATCC	ATGGTCTGGTGCAGTTGTCA
IL-22	CAACAGGCTAAGCACATGTCA	ACTGTGTCCTTCAGCTTTTGC
IFN-*γ*	GGCATTTTGAAGAATTGGAAAG	TTTGGATGCTCTGGTCATCTT
IL-8	AGACAGCAGAGCACACAAGC	AGGAAGGCTGCCAAGAGAG
IL-21	CTTGGTCCCTGAATTTCTGC	GCTGACTTTAGTTGGGCCTTC
IL-23R	GAAGACATGACACAGCCAACA	TCTGGAAGCAGGAAAAGACTG
TBX21	CCAACAATGTGACCCAGATG	AAAGATATGCGTGTTGGAAGC
GATA3	CTCATTAAGCCCAAGCGAAG	GTCTGACAGTTCGCACAGGA
FOXP3	ACCTACGCCACGCTCATC	TCATTGAGTGTCCGCTGCT
RORC	CAGCGCTCCAACATCTTCT	CCACATCTCCCACATGGACT
STAT1	GACTGAGTTGATTTCTGTGTCTGAA	ACACCTCGTCAAACTCCTCAG
STAT3	TCCTGAAGCTGACCCAGGTA	AGGTCGTTGGTGTCACACAG
STAT4	CAGGCTGAGTGGAGCCTTAT	CCACCTGCTCCAAAAACTTG
STAT5b	TATGCCACACAGCTCCAGAA	GTACAATATATGGCGGATGCAG
STAT6	GGTCGCAGTTCAACAAGGA	GTCCAGGACACCATCAAACC
AHR	CAACATCACCTACGCCAGTC	GCTTGGAAGGATTTGACTTGA
CYP1A1	TCCAAGAGTCCACCCTTCC	AAGCATGATCAGTGTAGGGATCT
CYP1B1	ACGTACCGGCCACTATCACT	CTCGAGTCTGCACATCAGGA
TIPARP	GGAAATTCTTCTGTAGGGACCA	GTTGGCTTCTTCAATCAATCG
PPIA	CATGGTGGCTCACTGTCTGT	GGCTGATCTTGACTCCTACCC
PGK1	TGCAAAGGCCTTGGAGAG	TGGATCTTGTCTGCAACTTTAGC
GUSB	CGCCCTGCCTATCTGTATTC	TCCCCACAGGGAGTGTGTAG

**Table 2:** Absolute concentration of cytokines (pg/mL) secreted by CD4^+^ T cells cultured under activation (anti-CD3/anti-CD28), Th17 polarizing conditions (TGF-*β*, IL-1*β*, IL-23), and with the AHR ligands FICZ, TCDD, and B[a]P measured by CBA (IL-17A, IL-9, IL-8, and IFN-*γ*) and ELISA (IL-22).

Cytokine	Time after activation (days)	Cytokine concentration (pg/mL), mean ± SEM, *n* = 15				
		CD4^+^ T cell culture conditions				
		Anti-CD3/anti-CD28	+Th17 polarizing condition	+FICZ (100 nM)	+TCDD (5 nM)	+B[a]P (100 nM)
IL-17A	1	1.25 ± 0	1.25 ± 0	1.25 ± 0	1.25 ± 0	1.25 ± 0
	2	1.25 ± 0	1.25 ± 0	4.11 ± 2.13	6.68 ± 5.43	4.10 ± 2.85
	3	1.25 ± 0	17.33 ± 5.63*	53.63 ± 13.52	41.09 ± 17.73	26.23 ± 15.92
	4	7.04 ± 3.59	81.89 ± 17.21*	131.39 ± 40.48	150.38 ± 46.28	163.37 ± 82.74
	5	279.86 ± 92.26	1629.89 ± 408.75*	620.48 ± 141.99^#^	607.14 ± 189.18	1382.26 ± 344.41

IL-22	1	n.a.	n.a.	n.a.	n.a.	n.a.
	2	n.a.	n.a.	n.a.	n.a.	n.a.
	3	13.84 ± 5.01	13.05 ± 6.89	32.52 ± 11.51	25.89 ± 12.96	17.15 ± 9.38
	4	n.a.	n.a.	n.a.	n.a.	n.a.
	5	2111.93 ± 462.2	506.47 ± 199.27^∗^	1585.37 ± 379.80^#^	1062.81 ± 454.26^#^	847.25 ± 200.17

IL-9	1	1.25 ± 0	1.25 ± 0	1.25 ± 0	1.25 ± 0	1.25 ± 0
	2	1.25 ± 0	1.25 ± 0	1.25 ± 0	1.25 ± 0	1.25 ± 0
	3	1.25 ± 0	7.81 ± 4.32*	20.53 ± 5.92	20.75 ± 8.43	14.63 ± 7.66
	4	1.25 ± 0	301.76 ± 70.10*	206.71 ± 54.25	314.55 ± 74.79	338.55 ± 137.18
	5	12.19 ± 6.31	2000.61 ± 386.25*	593.33 ± 121.46^#^	880.30 ± 242.76^#^	1309.34 ± 346.90

IL-8	1	1.99 ± 0.46	10.41 ± 2.26^∗^	13.22 ± 3.00	11.04 ± 2.31	9.76 ± 2.35
	2	3.40 ± 1.31	12.75 ± 4.03^∗^	20.88 ± 5.73	12.42 ± 1.84	10.94 ± 1.80
	3	7.94 ± 3.12	26.50 ± 5.75*	69.56 ± 9.73^#^	52.27 ± 11.72^#^	34.14 ± 8.16
	4	15.49 ± 7.33	57.44 ± 14.60	344.01 ± 57.41^#^	396.96 ± 79.42^#^	227.71 ± 61.40
	5	728.12 ± 272.04	652.82 ± 182.68	1739.35 ± 356.27^#^	2326.01 ± 325.16^#^	1278.65 ± 327.99

IFN-*γ*	1	9.99 ± 6.82	14.68 ± 9.53	15.56 ± 7.76	15.69 ± 8.96	13.91 ± 8.53
	2	10.16 ± 4.88	40.95 ± 4.97*	90.86 ± 19.71	83.73 ± 43.23	61.16 ± 30.68
	3	52.54 ± 15.31	161.24 ± 28.81*	381.06 ± 77.85	272.32 ± 65.30	193.48 ± 56.61
	4	122.46 ± 46.12	375.49 ± 93.63*	690.70 ± 134.00	712.15 ± 127.61	746.91 ± 188.30
	5	1007.50 ± 143.18	1104.35 ± 133.70	1219.11 ± 117.03	1137.87 ± 148.28	1303.77 ± 126.08

*represents significance compared to anti-CD3/anti-CD28, *P* < 0.05.

Mann-Whitney *U* test, and *n* = 15.

^#^represents significance compared to Th17 polarizing conditions, *P* < 0.05, Mann-Whitney *U* test, and *n* = 15.

n.a.: not analyzed.
